# Surgical management for squamous cell carcinoma of vulva

**DOI:** 10.11604/pamj.2016.24.145.8485

**Published:** 2016-06-15

**Authors:** Ayi Kossigan Amavi, Laurent Kouadio, Komlan Adabra, Kodjo Tengue, Fouad Tijami, Abdelouahed Jalil

**Affiliations:** 1 Service de Chirurgie Oncologique, Institut National d'Oncologie, Rabat, Maroc; 2 Chirurgie Générale, Viscérale, Chirurgie Oncologique, Clinique Médico-chirurgicale, Centre Hospitalier Universitaire Sylvanus Olympio, Lomé, Togo

**Keywords:** Locally advanced, cancer, vulva, surgery

## Abstract

To analyze our surgical management and the result of squamous cell carcinoma (SCC) of vulva. Retrospectively, we collected 38 cases of SCC; 17 cases of them were early SCC and 21 cases were locally advanced. The patients underwent primary surgery. The survival was estimated using Kaplan-Meier analysis and the log rank test. The mean age was 60.78 years. Total vulvectomy was performed in all patients. Superficial and deep incision of bilateral inguinal lymphadenectomy was performed by separates incisions for SCC infiltrating more than 1mm. The average tumor size was 53 mm (10 to 140mm). Morbidity was 42.1%. Lateral resection margin ≥8mm was obtained in 57.1%. Eighteen patients benefited from adjuvant radiotherapy. The follow-up median was 19.4 months (6 to 61.5 month) with 05 recurrences in 12 months. The survival using the Kaplan-Meyer analysis at 5 years, was 62.1% (71.2%N^-^ vs 46.7%N^+^; p = 0.13). We identified two groups for locally advanced vulva cancer. Primary surgery keeps its place. Neo adjuvant radio chemotherapy followed by surgery is the alternative treatment for locally extensive lesions.

## Introduction

quamous cell carcinoma (SCC) of vulva is a rare cancer. It represents 3-5% of gynecological cancers with an incidence of 2 per 100 000 women/year [1]. It affects women of the 6th decade. Surgery remains the cornerstone of its management. The treatment is well codified for the early stages whereas locally advanced carcinomas (stage III and IV) may be difficult to manage. The morbidity of this inguinal vulvar surgery may be important. It sometimes includes durable and disabling sequels. Also, radiotherapy and/or chemotherapy progressively find more important place in the treatment of localized SCC of vulva. In the light of literature data, we present our series of 38 cases of vulvar SCC, discuss our result and therapeutic indications especially in locally advanced stages which management is not yet codified.

## Methods

We undertook a retrospective descriptive study between 1^st^ January 2007 and 31^th^ December 2010 in the J surgery department of the National Institute of Oncology (INO) in Rabat. We collected 38 cases of SCC of vulva, including early and advanced stages, managed by first surgery. Anatomo-pathological results of biopsy samples made before surgery revealed 36 cases of infiltrating SCC and 02 cases of in situ lesions extended to the vulvar. Chest X-ray, inguinal ultrasound and/or abdominopelvic scan were the preoperative radiological assessment performed for locally advanced lesions. With data collected, we classified our patients into two groups according to the clinical status of tumor: Group 1: early SCC of vulva (17 cases); T ≤ 2cm or T > 2cm, unifocal or multifocal, localized to an hemivulva, and without clinically node invasion; Group 2: locally advanced SCC of vulva (21 cases); T > 2cm taking at least one hemivulva with sometimes local extension to the perineum, vagina, urethra, anus and beyond. The data analyzed were age, clinical features of vulva lesions with the extension assessment allowing the TNM/FIGO classification 2009; performed the surgical procedures, length of postoperative stay, morbidity, anatomo-pathological findings of the surgical specimen, and adjuvant therapy. In order to study overall survival (OS), we tried to contact every patient. The survival was estimated using Kaplan-Meier analysis and the log rank test. The difference was considered significant for p ≤ 0.05. Self evaluation of one's image and impact on sexual life were not appreciated.

## Results

### Clinical description

The mean age of patients was 60.78 years (32 to 91 years). The size of the tumor, its location and local extension are summarized in Table 1. Ulcerated and/or burgeoning aspects with local secondary infection were found in 33 cases (86.84%). Other clinical aspects were whitish flat lesions (3 cases) and vulvar nodules secondary to recurrence (02 cases). No distant metastases were noted.

**Table 1:** The location of the lesion, it's local extension, and inguinal node invasion depending on the tumor size

	Location of lesion					Local extension (13 cases)			N(+) =21 cases		
	HL	SL	Clitoris	HV	T	Vagina	Urethral Meatus	perineum	R	L	R/L
≤2cm			1	0	10	0	0	0	2	2	0
>2cm	17	2	2	7	28	6	5	2	6	7	4

HL: High lip, SL: Small lip, HV: hemivulva, T: total; R: right, L:left; N(+): inguinal node

### Surgery

The first total vulvectomy was performed in all patients. The resection was sometimes extended to the urethral meat (5 cases), to vagina (6 cases), and/or to perineum (2 cases). The length of the urethra resected was less than 1cm in 2 cases, one case between 1 cm and 1.5 cm, and more than 1.5 cm in 2 cases. No anal sphincter resection was performed. Lymphadenectomy was associated in all cases of infiltrating SCC up to 1 mm (28 cases). Technically, the inguinal dissection was bilateral superficial and deep. It was done by bilateral inguinal incision separated from the vulvectomy incision except in one patient who had an inguino-vulvar incision due to the extension of the tumor on the mount of Venus. The Lymphadenectomy was delayed and performed 3 weeks after vulvectomy in 03 cases of superinfected tumors for which Ro resection remained equivocal. Search of sentinel node was made in 01 case for a 1cm tumor. After inguinal lymphadenectomy, protection of femoral vessels by transposition of the sartorius muscle was performed, followed by a suction drain. The mean postoperative stay was 10.8 days (5 to18 days). Twenty-five patients returned home before the 10^th^ day. In the immediate postoperative period, 16 patients (42.1%) presented with morbidities that we classified according to the “Common Terminology Criteria for Adverse Events version 4.0 2010” Table 2.

**Table 2:** Surgical morbidity according to «Common Terminology Criteria Adverse Events version 4.0 2010»

Surgical morbidity	Total	Grade 1	Grade 2	Grade 3
Superinfection/ desinuty of suture	6	2	4	
seroma / lymphodema	6	3	3	
Delay healing	10	7	3	
urinary incontinence	2	2		
Dysuria	3	3		

### Anatomo-pathological findings and pTNM classification

Twenty-seven (27) patients were classified at least pT2. We had 7 cases of stage I (VIN 01case, pT1a 02 cases and pT1b 04 cases), 16 cases of stage II, and 15 cases of stage III (pIIIa 4 cases, pIIIb 3 cases and pIIIc 8 cases). The average tumor size was 53 mm (10 to 140 mm). The margin of lateral and deep resection is presented in Table 3. The number of lymph nodes examined has varied between 3 and 21 per dissection. The size of positive nodes varied between 10mm and 40mm.

**Table 3:** Lateral and deep margin of resection (mm) on fixed tissue depending on the tumor size (cm)

	LM		DM		NS
	<8mm	≥8mm	<5mm	≥ 5mm	-
≤2cm(10)	0	6	2	4	4
>2cm(28)	7	16	9	14	5

LM: lateral margin (<8mm and ≥ 8mm); DM: deep margin (<5mm and ≥ 5mm) NS: no specificed

### Adjuvant treatment

Eighteen patients were admitted to radiotherapy for adjuvant therapy. Indications were: margin of lateral resection < 8mm, margin of deep resection < 5mm, lymph node involvement or unspecified margin of resection. Six complications of radiotherapy were found: 5 cases of vaginal sclerosis and one case of dyspareunia. No patient received chemotherapy.

### Follow up

**Figure 1:**
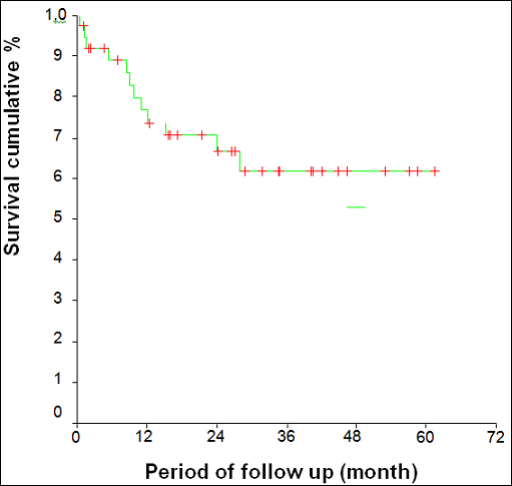
The curve of overall survival

Median follow-up was 19.4 months (6 to 61.5 months). After 12 months follow-up, we observed 05 cases of recurrence: 04 recurrences to inguinal lymph nodes and 01 local recurrence (Table 4). The 5-years survival rate was 62.1% (Figure 1) (71.2% N^-^ vs 46.7% N^+^; p = 0.13). The 5-year overall survival for stage I, II, and III were respectively 71.4%, 70% and 46.7% (p = 0.32). There was a significant difference (p = 0.01) between 5 years overall survival for patients ages < 60 years (82.4%) and ≥ 60 years (38.6%)

**Table 4:** Recurrence after 12 months, clinical and therapeutic characteristics according to the place of recurrence

	Vulvar recurrence (01)	Inguinal recurrence (04)			
	patient 1	Patient 2	Patient 3	Patient 4	Patient 5
Inguinal lymphadenectomy	Yes	No	No	No	Yes
N+/N	5/10(R) 6/10 (L)	-	-	-	0/21 (R) 2/11 (L)
Tumor size	130mm	75mm	56mm	80mm	40mm
Margin(L/D) mm	8/4	4/3	20/5	20/4	15/9
Radiotherapy	No	No	No	No	Yes

N+/N: positive node / total nodes removed Margin (L/D): lateral margin / deep margin ( millimeters)

## Discussion

SCC of vulva preferentially affects the elderly women. The main location is the labia majora. In 10% of cases, the lesions are largely extensive and the primary site is difficult to determine [2]. The proportion of stage III and IV cancers in our series (39.5%) corresponds to the literature datas (30% to 46.7%) [3, 4]. Contrarily to early SCC, the concept of advanced SCC is not well defined. In the classification of FIGO, stage III or IV lesions are considered as locally advanced SCC. However, a tumor measuring 2 cm, without extension to the distal urethra and/or anus but invading nodes is considered stage III for FIGO 2009. In our series, we considered local extension of the tumor. In therapeutic codification, vulvar surgery is, when it is possible, the best way to relieve the patient and avoid local evolution. The difficulties of the surgical management of locally advanced SCC of vulva are not only technical, but also take in account morbidity problems and the care for oncological margin Ro resection. It is therefore necessary to make a review on this affection, regarding the therapeutic choices in situations where the lesions are highly evolved. During the last decade, a minimally invasive by first partial vulvectomy or local excision is admitted for surgery of early SCC [5, 6]. Early infiltrating SCC (> 1mm) which extension did not exced 10 mm from the median line, may yet benefit preoperatively of inguinal ultrasound and a dual technology (isotopic and colorimetric) sentinel node detection. In our series, early SCC of vulva underwent radical vulvectomy with bilateral inguino-femoral lymphadenectomy (LIF) for lesions infiltrating more than 1mm. In our study, it was an excessive surgery for such unifocal tumors which size was inferior to 2 cm. In case of less mutilating resection with a macroscopic margin of 1 cm to 2 cm, no significant difference was found in terms of recurrence and mortality, compared to radical vulvectomy [7]. Lymphadenectomy was the only way referenced that could give us full histological analysis of the inguinal nodes. Its complications are frequent and often disabling [5, 6, 8]: suture dehiscence (13-38%), seroma (12-40%), secondary infection (22-57%) and lymphedema (13-53%). As economical consequence, there is an increase of the average length of stay.

Locally advanced SCC of vulva requires first extensive surgery. It is recommended to obtain a macroscopic surgical margin superior to 2 cm [4, 8, 9] in order to minimize the probability of obtaining a pathological margin under 10 mm. Authors [4, 9, 10] have reported that histological margin < 8mm was accompanied with a recurrence rate of 23% to 52.6%. In our series, we noted 34% of extensive surgery with resection of the distal urethra, vagina and perineum. The margin of lateral resection ≥8mm (only 57.1% in our series) was more difficult to obtain in locally advanced SCC. Direct morbidity consisted in disunity of the suture by tension or superinfection, delayed wound healing, and urinary incontinence. Despite a complete inguinal lymphadenectomy, the risk of inguinal recurrence still exists. After 12 months follow-up, the recurrence rate in our series was 13.2%. Risk factors of recurrence in our study were: tumor size ≥40 mm, lack of inguinal lymphadenectomy, lack of adjuvant radiotherapy, and refusal of treatment (in 04 elderly patients). The Spanish Society of Obstetrics and Gynecologist (SEGO) considers 40 mm the limit between tumors with high and low risk [11]. The Risk factors for local and inguinal recurrence are tumor size (≥35mm), margin of resection (≤15mm), degree of invasion (4mm) and initial nodal involvement [10]. In the literature, the overall survival is 70% to 93% (N^-^) and 25% to 41% (N^+^) [12, 13]. In our series, primary surgery followed by radiation therapy, 5-years overall survival was 62.1% (71.2% N^-^ vs 46.7% N^+^). Our survival rate was influenced by nodal involvement and the age over 60 years that are deleterious factors. The age group from 60 years old accounted for 50% of our series. Gonzalez-Bosquet [14] of 330 patients, had reported 44% of inguinal recurrence, and 67% of these recurrences died within 2 years. The management of the locally advanced SCC of vulva remains complex. This management must be as least as morbid possible without compromising the oncological outcome and improving the survival. Treatment modalities combine surgery, radiation and chemotherapy. The analysis of our series allowed us to note two groups of patients with locally advanced SCC of vulva. The locally advanced SCC of vulva affecting vaginal or anal without urethral extension. They can benefit from a first surgery with adjuvant radiation therapy especially in young patients (less than 60 years, 5-years overall survival was 82.4% in our series; locally advanced SCC of vulva SCC with anal or urethral extension, even beyond. Apart from the immediate morbidity of surgery, the long-term difficulties are related not only to relapse but also the physical and psychological impact of surgery on these patients. Concomitant chemoradiation therapy represents the first new therapeutic approach [15]. The use of surgery should be evaluated again in order to avoid ultra radical resection or pelvic exenteration. This new approach would be quite interesting in view of the good results in anal, cervix and rectum cancers.

## Conclusion

The optimal management of advanced vulva cancer is complex and requires the use of a multi-modality approach. One of the problems of this management is the low number of cases managed in different series. The first surgery keeps an important place. For large lesions, chemoradiation with or without surgery is the new therapeutic approach, in order to limit morbidities.

### What is known about this topic

- Surgical approach is the first method for the treatment of vulva cancer;
- Locally advanced vulva cancer underwent chemoradiation follow by surgery. In others situations, it suggested to use chemotherapy induction;
- The management for advanced vulva cancer are not yet clear in the literature.

### What this study adds

- That is one of the first surgical studies with interesting results, which are the key for orienting a codified advanced approach for treating cancer of the vulva. It identified two group of locally advanced vulva cancer.
